# Acceptability and Usability of a Socially Assistive Robot Integrated With a Large Language Model for Enhanced Human-Robot Interaction in a Geriatric Care Institution: Mixed Methods Evaluation

**DOI:** 10.2196/76496

**Published:** 2025-08-01

**Authors:** Lauriane Blavette, Sébastien Dacunha, Xavier Alameda-Pineda, Daniel Hernández García, Sharon Gannot, Florian Gras, Nancie Gunson, Séverin Lemaignan, Michal Polic, Pinchas Tandeitnik, Francesco Tonini, Anne-Sophie Rigaud, Maribel Pino

**Affiliations:** 1 Institut national de la santé et de la recherche médicale - Optimisation thérapeutique en pharmacologie OTEN U1144 Université Paris Cité Paris France; 2 Assistance Publique - Hôpitaux de Paris, Hôpital Broca Centre Mémoire de Ressources et Recherches Île-de-France-Broca Service gériatrie 1&2 Paris France; 3 Broca Living Lab Hôpital Broca Paris France; 4 Institut national de recherche en sciences et technologies du numérique de l'Université Grenoble Alpes Grenoble France; 5 Interaction Lab, Mathematical and Computer Sciences Heriot-Watt University Edinburgh United Kingdom; 6 Faculty of Engineering Bar-Ilan University Bar Ilan Israel; 7 ERM Automatismes Carpentras France; 8 PAL Robotics Barcelona Spain; 9 Czech Institute of Informatics, Robotics and Cybernetics Czech Technical University in Prague Prague Czech Republic; 10 Department of Information Engineering and Computer Science University of Trento Trento Italy

**Keywords:** socially assistive robot, hospital environment, gerontology, older adults, informal caregivers, acceptability, usability, large language model, human-robot interaction

## Abstract

**Background:**

Socially assistive robots (SARs) hold promise for supporting older adults (OAs) in hospital settings by promoting social engagement, reducing loneliness, and enhancing emotional well-being. They may also assist health care professionals by delivering information, managing routines, and alleviating workload. However, their acceptability and usability remain major challenges, particularly in dynamic real-world care environments.

**Objective:**

This study aimed to evaluate the acceptability and usability of a SAR in a geriatric day care hospital (DCH) and to identify key factors influencing its adoption by OAs and their informal caregivers.

**Methods:**

Over the course of 1 year, 97 participants (n=65, 67%, OA patients and n=32, 33%, informal caregivers) took part in a mixed methods evaluation of ARI, a socially assistive humanoid robot developed by PAL Robotics. ARI was deployed in the waiting area of a geriatric day care robot in Paris (France), where it interacted with users through voice-based dialogue. After each session, participants completed 2 standardized assessments, the Acceptability E-scale (AES) and the System Usability Scale (SUS), administered orally to ensure accessibility. Open-ended qualitative feedback was also collected to capture subjective experiences and contextual perceptions.

**Results:**

Acceptability scores significantly increased across waves (wave 1: mean 15.4/30, SD 5.81; wave 2: mean 20.9/30, SD 5.25; wave 3: mean 22.5/30, SD 4.23; *P*<.001). Usability scores also improved (wave 1: mean 47.9/100, SD 24.18; wave 2: mean 57.4/100, SD 22.46; wave 3: mean 69.3/100, SD 16.03; *P*<.001). A strong positive correlation was observed between acceptability and usability scores (r=0.664, *P*<.001). Qualitative findings indicated improved ease of use, clarity, and user satisfaction over time, particularly following the integration of a large language model (LLM) in wave 2, leading to more coherent, natural, and context-aware interactions.

**Conclusions:**

Successive system enhancements, most notably the integration of an LLM, led to measurable gains in usability and acceptability among patients and informal caregivers. These findings underscore the importance of iterative, user-centered design in deploying SARs in geriatric care environments.

**Trial Registration:**

Approved by the French national ethics committee (CPP Ouest II, IRB: 2021/20) as it did not involve randomization or clinical intervention

## Introduction

The rapid aging of the population worldwide is one of the major demographic challenges of the 21st century. According to United Nations projections, the number of people aged 65 years and over worldwide is expected to increase from 727 million in 2020 to over 1.5 billion by 2050 [1]. This demographic evolution is accompanied by a growing need for long-term care and increased use of specialized services, particularly in geriatric institutions [2]. Consequently, geriatric institutions will face significant challenges in managing a rising number of residents, many of whom present multiple chronic conditions that require comprehensive and personalized care [3]. In response to this societal challenge, the adoption of innovative technologies and, in particular, socially assistive robots (SARs), is increasingly considered a complementary solution to improve the well-being and autonomy of older adults (OAs) [4,5]. SARs are robotic entities designed to interact with humans in socially and emotionally engaging ways. According to Dautenhahn [6], a SAR is “a robot that can interact with humans in a social context, while possessing communication and learning capabilities that mimic, to some extent, human behaviors.” These robots integrate artificial intelligence (AI) and natural language processing to a certain extent, enabling them to interact more naturally with users, leading to enhanced engagement and adaptation to different social contexts [7]. Within geriatric institutions, SARs have the potential to alleviate caregiver workloads, provide cognitive and social stimulation for patients, and assist with daily tasks [8]. However, for these innovations to be successfully implemented and fully beneficial, 2 critical ergonomic dimensions must be addressed: their acceptability and their usability in human-robot interaction (HRI).

The effectiveness of SARs in geriatric institutions largely depends on the quality of HRI [5,9]. Acceptability refers to how willing users—including patients, their informal caregivers, and health care professionals—are to adopt these new tools. This willingness is shaped by their beliefs, needs, and trust in the system [10,11]. Usability, in this context, refers to the extent to which a system supports users in accomplishing their tasks effectively, efficiently, and with satisfaction, within a defined environment. Beyond the system’s ergonomic characteristics, such as interface layout, feedback modalities, and physical interaction, it also encompasses aspects such as learnability, error tolerance, and cognitive demand, which are particularly critical when designing for OA populations. [12-14].

Despite their potential, SARs still face significant limitations regarding the quality of HRI and user engagement with the technology. Studies highlight that SARs often struggle to convey emotions effectively, understand the context, and predict users’ behaviors accurately [15-17]. These shortcomings can lead to rigid and unnatural communication, hindering the robot’s ability to interpret human intentions and emotions [18]. Additionally, SARs often lack adaptability to individual preferences, which can result in a gradual decline in the user’s interest and engagement [19]. These challenges highlight the importance of designing HRIs that are intuitive, customizable and aligned with the expectations of OAs to enhance both acceptability and successful integration into geriatric institutions. For example, OAs expect robots to use clear, slow speech; maintain a polite, emotionally supportive tone; minimize technical vocabulary; and favor voice-based over touchscreen-based interaction, all of which have been repeatedly identified as essential for promoting acceptance and usability among OAs [5,19-23].

To overcome these limitations, recent research has explored the integration of large language models (LLMs) into SARs to enhance their communication and interaction capabilities [24-26]. LLMs allow for greater conversational flexibility, improved comprehension of complex requests, and enhanced contextual coherence in responses [27,28]. However, these advancements also introduce new challenges, including biases in generated responses, lack of transparency in robot decision-making, and difficulties in real-time interaction management [29,30].

Given the cognitive and physical limitations often present in OAs, SARs must feature intuitive and user-centered HRIs to maximize their effectiveness [20]. SAR acceptability encompasses not only perceived ease of use but also alignment with user needs, including preferences related to the communication style, autonomy, affective support, and interaction dynamics [10,31-35]. Among OAs, factors such as low digital literacy, distrust of technology, and sensory or cognitive impairments can influence acceptance [21,36]. Barriers to adoption may include reluctance regarding the robot’s appearance, the communication style, or ethical concerns about reduced human interaction [11].

Simultaneously, usability focuses on how efficiently, effectively, and satisfactorily a system can be used to perform expected tasks [13,14]. In geriatric institutions, usability considerations extend to physical ergonomics (eg, size, weight, tactile interfaces, voice interaction), user safety, and emotional comfort [37]. Evaluating SAR usability through standardized methods, such as the System Usability Scale (SUS), helps obtain insights into adoption barriers, assess the ease of operation, and guide iterative technological improvements [12,38]. These aspects emphasize the necessity of a user-centered approach tailored to the specific needs of elderly users [20].

Several studies have investigated the acceptability and usability of SARs by OAs in real-world contexts, including institutional settings, such as nursing homes and hospitals [19,37,39]. However, many of these evaluations rely on scripted interactions or exclude contextual constraints found in routine care environments. Although increasing attention has been paid to the perceptions of professional caregivers, relatively few studies have examined the opinions of informal caregivers (family or friends), despite their role in health care decision-making and daily care activities [21,36]. These gaps highlight the need for in situ evaluations that incorporate the perspectives of both patients and informal caregivers, while considering institutional realities.

To tackle these challenges, empirical research is essential to assess the acceptability and usability of SARs in real-world conditions. This study aimed to evaluate the performance, acceptability, and usability of a SAR among patients and their informal caregivers in a geriatric day care hospital (DCH) in Paris (France). It also aimed to identify the factors that facilitate or hinder SAR adoption in geriatric institutions. The findings will provide deeper insights into the expectations and concerns of OAs and their informal caregivers regarding the use of SAR in health care, while offering a set of recommendations to guide future technological and ergonomic advancements in this field.

## Methods

### Participants

This study involved 2 populations: OA patients attending consultations at a geriatric DCH and their informal caregivers.

*Inclusion criteria* for patients were (1) being ≥60 years old, (2) having a Mini-Mental State Examination (MMSE) score above 10 (indicating the absence of severe cognitive impairment; based on standard thresholds, scores of 25-30 are considered normal, 21-24 as mild, 10-20 as moderate, and below 10 as severe) [40], and (3) not exhibiting symptoms of altered reality and understanding and speaking French fluently. Informal caregivers were family members or friends (primarily spouses or children) aged ≥18 years, accompanying the patient and speaking French fluently.

No *exclusion criteria* were applied based on gender, socioprofessional backgrounds, or ethnicity.

#### Recruitment

Recruitment was carried out using the DCH database, and participants were prescreened prior to enrollment, contacted over the phone, and invited to participate in the study the day of their next consultation. An information letter was sent by post and informed consent was collected onsite.

#### Setting

The study was conducted between May 2023 and July 2024 in the geriatric DCH of the French Memory Clinic of a geriatric hospital in Paris (France). The DCH provides specialized outpatient care for OAs with physical or cognitive impairments, offering a wide range of consultations, including neurology, oncology, cardiology, psychiatry, and memory assessments. Three waves of data collection were carried out during this period, with a different sample of participants included in each iteration.

### Study Design

A mixed methods design was used, combining qualitative and quantitative methods to provide an in-depth understanding of the acceptability and usability of a SAR in a geriatric institution. Mixed methods are particularly suited to exploring complex phenomena in the health and social sciences, as they enable the integration of complementary perspectives and strengthen the validity of results [41,42]. By combining quantitative rating scales with semistructured interviews, this approach identifies both general trends and nuances specific to users’ perceptions [43,44]. Mixed methods also enable data triangulation, essential for understanding factors that influence SAR adoption in real-world contexts [45]. This approach, widely recognized in health service evaluation, guarantees a richer, contextualized analysis of the dynamics at play [46,47].

### Materials

#### ARI Robot

**Figure 1:**
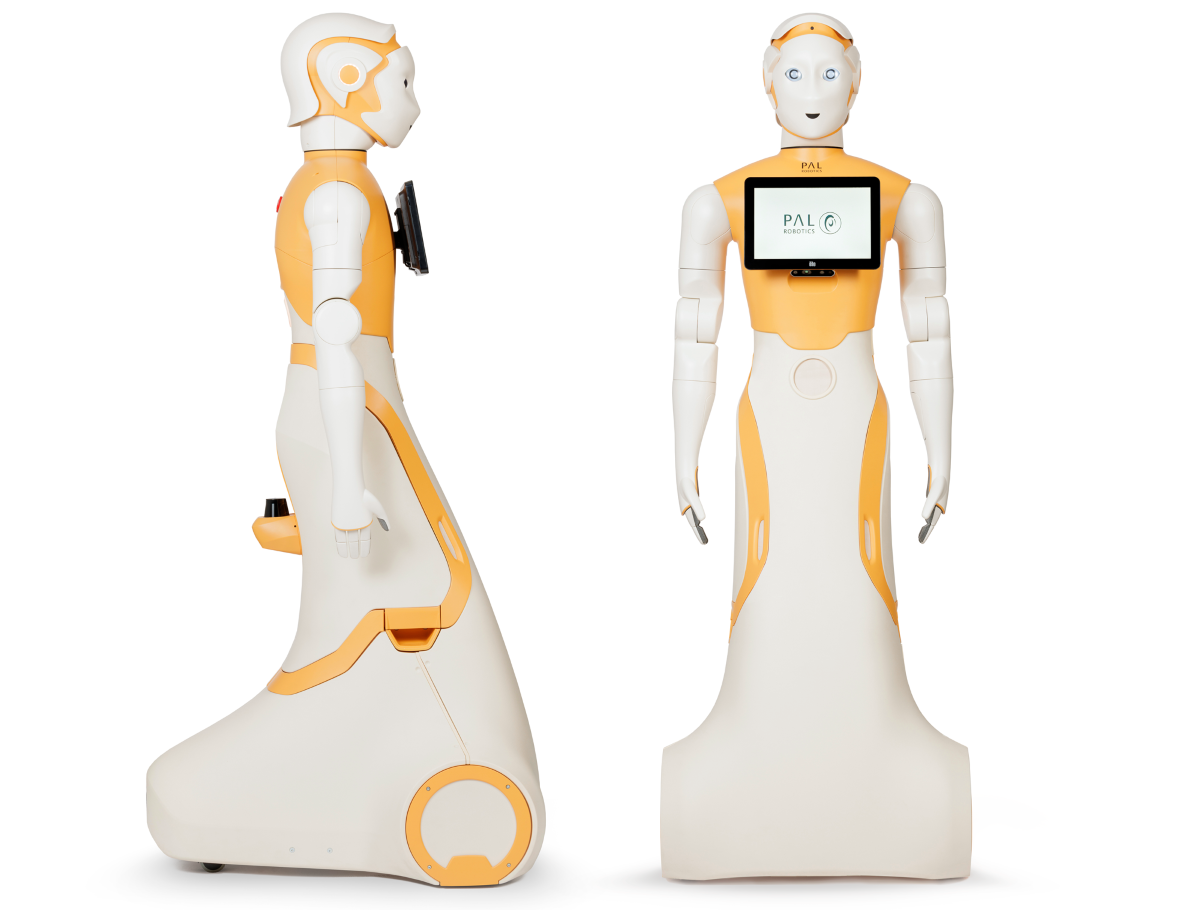
Photos of the ARI robot: front and side (photo credit: PAL Robotics).

The SAR used in this study was ARI, developed by PAL Robotics (Spain) [48]. The robot is 1.65 m (5 ft 5 in) tall and weighs 50 kg (Figure 1). The robot moves by rolling and is equipped with articulated arms that are not designed for load bearing. Its interface includes a touchscreen located on the torso for dialogue transcription, animated eyes with a gaze-tracking module, luminous ears, and an emergency stop button on the back of the robot. ARI supports autonomous operation for 8-12 hours prior to recharging. It is equipped with wired and wireless connectivity capabilities for flexible integration into a variety of environments. For visualization, ARI is equipped with 3 wide-angle cameras positioned on the head, chest, and back, giving it a wide and versatile view of its surroundings. In terms of audio, the robot features 4 microphones, facilitating voice recognition and the efficient capture of ambient sounds.

For this study, the ARI robot was programmed with a set of modules explicitly developed as part of the European H2020 SPRING (Socially Pertinent Robots in Gerontological Healthcare) project described by Alameda-Pineda et al [49]. Among these modules, the conversational system was initially developed before recent LLM advances (eg, ChatGPT), relying on a “traditional” modular architecture that combines retrieval-based responses, rule-based intent handling, and open-domain generation (wave 1) [50]. To take advantage of LLMs’ abilities for solving complex language-related tasks, the conversational system was redeveloped (wave 2) and refined (wave 3) with an LLM-based architecture, based on the Vicuna model with 13 billion parameters (Vicuna-13b-v1.5 [51]). For deployment in hospital settings, it was adapted to function offline and integrated with a custom prompt targeting health care–related scenarios in French. This design ensured safe use without requiring an internet connection, while enabling more coherent and context-sensitive interactions with patients and informal caregivers.

At the time of the study, ARI had not yet been commercially deployed in hospitals or long-term care institutions beyond the scope of research projects. Its use in this evaluation should therefore be considered as part of an exploratory, precommercial experimentation phase within controlled health care settings.

#### Introduction of ARI’s Capabilities

The SAR was deployed in the waiting area of a geriatric DCH to welcome and assist patients and their informal caregivers. Although participants were free to interact spontaneously with the robot, the researcher introduced 5 illustrative use cases at the beginning of the session to showcase the robot’s potential functionalities: (1) greeting and welcoming the user, (2) recalling hygiene and infection prevention procedures, (3) providing information about the consultation procedure, (4) offering orientation and guidance about hospital services, and (5) providing entertainment activities. These examples were intended to guide participants’ understanding of the robot’s capabilities without restricting the content of their interactions.

### Ethical Considerations

The study was approved by the French National Ethics Committee Comité de Protection des Personnes, CPP Ouest II, Maison de la Recherche Clinique-CHU Angers (Institutional Review Board [IRB]: 2021/20) and complied with the General Data Protection Regulation (GDPR; DPO: 20210114153645, AP-HP register). It did not involve randomization or clinical intervention. Informed consent was obtained from all participants onsite. Participants were informed that they could stop their participation at any time. The original consent covered the secondary analysis without further consent. Participant data were anonymized. No compensation was provided to the participants.

### Assessment Scales

To assess the acceptability and usability of the ARI robot in a DCH, we used 2 standardized scales:

- The Acceptability E-scale (AES): Acceptability was assessed using the AES, French version [52]. Acceptability is defined as the psychological determinants that shape an individual’s intention to use a technology prior to any direct experience with the system. The AES scale comprises 6 items rated on a 5-point Likert scale, yielding a total score ranging from 6 to 30. For marketable products, the AES’s acceptability threshold is set at 25.81/30.
- The System Usability Scale (SUS): Usability was assessed using the SUS [53,54]. Usability refers to the degree to which a system can be used by specified users to achieve specific goals with effectiveness, efficiency, and satisfaction in a specified context of use. Ease of use influences user performance and satisfaction, while acceptability determines actual usage [55]. The SUS consists of a 10-item scale designed to assess the overall usability of a system, generating an overall score out of 100, where a higher score reflects better usability. For this scale, experimenters are asked to respond to statements on a 5-point Likert scale, with ratings ranging from “strongly disagree” to “strongly agree.” The usability threshold for marketable products is 72/100 for this scale.

The adapted versions of both scales used in this study are provided in Multimedia Appendix 1. For each scale, participants were invited to comment on their choices in a discussion with the researcher.

### Assessment Procedure

Each session (~45 minutes) took place in a dedicated room. After free interaction with the robot, participants completed 2 validated instruments: the AES and the SUS. Both were administered orally and supported by open-ended discussions to elicit qualitative data. Finally, participants were accompanied back to the waiting area.

### Data Analysis

To ensure a comprehensive understanding of the research topic, both qualitative and quantitative data were collected. Each session was audio-recorded and subsequently transcribed for analysis.

Descriptive statistics (means, SDs, and percentages) were used to describe the sample characteristics and the scores obtained on the AES and SUS.

To assess statistical differences between waves and between groups, several statistical tests were performed. First, Kruskal-Wallis tests were used to compare multiple groups, and Mann-Whitney tests were used to compare 2 scores or 2 groups. Shapiro-Wilk tests were used for normality assumption. When significant differences were found, Tukey post hoc tests were conducted to further explore the data. *P*<.05 was considered statistically significant in all analyses.

Qualitative data were analyzed using inductive thematic analysis [56], allowing themes to emerge from the transcripts of the interviews with the researcher.

### System Evolution for Each Experimental Wave

**Figure 2:**
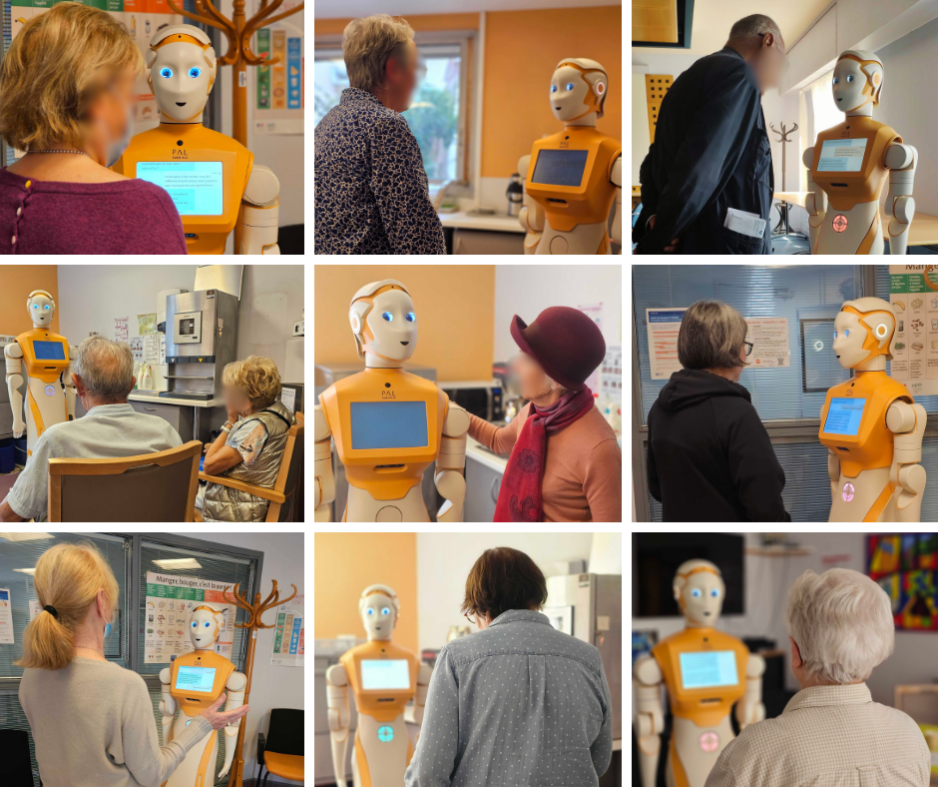
Illustration of experiments.

To evaluate the evolution of the ARI robotic system with the help of feedback from participants, we carried out system updates. Over the evaluation period, the ARI robot received 2 updates, resulting in 3 waves of experimentation (Figure 2). Table 1 shows the overall differences in system performance over the 3 test waves, and the full list is described in SPRING Deliverable D1.6 [57].

**Table 1:** System evolution based on participants’ feedback.

Aspect	Wave 1 (May-July 2023)	Wave 2 (September-December 2023)	Wave 3 (March-May 2024)
Robustness of the robot’s responses	Basic diagnostic tools to prevent operational failures; first version of a modular dialogue system	Improved accuracy of diagnostic tools; reduced memory/computational load for vision modules; integration of an extended LLM^a^	Enhanced general system stability; improved speech recognition robustness
Feature display and interactive functionalities	Display of dialogues’ transcription on the robot’s screen	Optimized display output; improved display model for transcribed speech	Conversation start/end display; operator speech control; refined LLM prompt tailored to a hospital context
Perception and tracking	Depth estimation of the environment; person tracking; facial identification modules	Integration of a module for associating vocal input to individual users (Ecapa model); optimization of facial recognition processing time	Addition of head movements enabling the robot to follow participants’ gaze; optimization of gaze estimation; improved audiovisual tracking; enhanced speaker identification (Ecapa2 model); improved voice activity detector

^a^LLM: large language model.

## Results

### Participants’ Sociodemographic Data

#### Descriptive Statistics

Over the 3 experimental waves, 110 participants agreed to take part in the study. Of these, only ninety-seven (88.2%) completed the experiment. Incomplete robot assessment sessions occurred when participants were called away for their scheduled medical appointments. The results presented reflect the feedback from these 97 participants across the 3 experimental waves (wave 1: n=14, 14.4%; wave 2: n=43, 44.3%; wave 3: n=40, 41.3%). Table 2 shows the sociodemographic data for the participants in each wave. Participants were categorized as patients (P) or informal caregivers (IC).

**Table 2:** Sociodemographic data for each experimental wave.

Profile and characteristics			Wave 1 (n=14)		Wave 2 (n=43)		Wave 3 (n=40)		Total (N=97)
**P^a^**									
	Participants, n/N (%)	11/14 (78.6)		30/43 (69.8)		24/40 (60.0)		65/97 (67.0)	
	Males, n/N (%)	2/11 (18.2)		9/30 (30.0)		6/40 (25.0)		17/65 (26.2)	
	Females, n/N (%)	9/11 (82%)		21/30 (70.0)		18/40 (75.0)		48/65 (73.8)	
	Age (years), mean (SD)	78.4 (7.1)		78.3 (6.6)		81.2 (6.4)		79.4 (6.7)	
	Education (years), mean (SD)	13.6 (2.1)		12.9 (3.5)		11.4 (3.6)		12.5 (3.4)	
	MMSE^b^ score, mean (SD)	27.8 (1.9)		25.2 (4.5)		25.5 (3.9)		25.7 (4.1)	
**IC^c^**									
	Participants, n/N (%)	3/14 (21.4)		13/43 (30.2)		16/40 (40.0)		32/97 (33.0)	
	Males, n/N (%)	1/3 (33.3)		5/13 (38.5)		9/16 (56.3)		15/32 (46.9)	
	Females, n/N (%)	2/3 (66.7)		8/13 (61.5)		7/16 (43.7)		17/32 (53.1)	
	Age (years), mean (SD)	75.3 (11.6)		63.9 (19.4)		67.9 (12.9)		67 (15.7)	
	Education (years), mean (SD)	12.0 (3.0)		15.0 (0)		13.2 (4.1)		13.8 (3.2)	
	MMSE score, mean (SD)	N/A^d^		N/A		N/A		N/A	

^a^P: patients.

^b^MMSE: Mini-Mental State Examination.

^c^IC: informal caregivers.

^d^N/A: not applicable.

Sociodemographic data analysis using nonparametric tests (justified by significant Shapiro-Wilk tests indicating nonnormal distributions: *P*<.001 for socioeducational level, *P*=.008 for MMSE score, and *P*≤.003 for age across waves 2 and 3) revealed no significant difference in terms of age across the 3 waves (*χ*²_2_=0.388, *P*=.82) and no significant difference in the socioeducational level (*χ*²_2_=3.330, *P*=.19), suggesting a relatively homogeneous distribution of these characteristics among the groups. Furthermore, no significant difference was observed regarding the MMSE scores of patients across waves (*χ*²_2_=4.064, *P*=.13).

A correlation analysis was carried out between AES and SUS scores in order to explore the consistency of participants’ responses across the 2 scales. The results revealed a strong positive correlation between the 2 variables (r=0.664, *P*<.001), indicating that higher acceptability scores are associated with higher usability scores.

### Acceptability E-Scale

**Figure 3:**
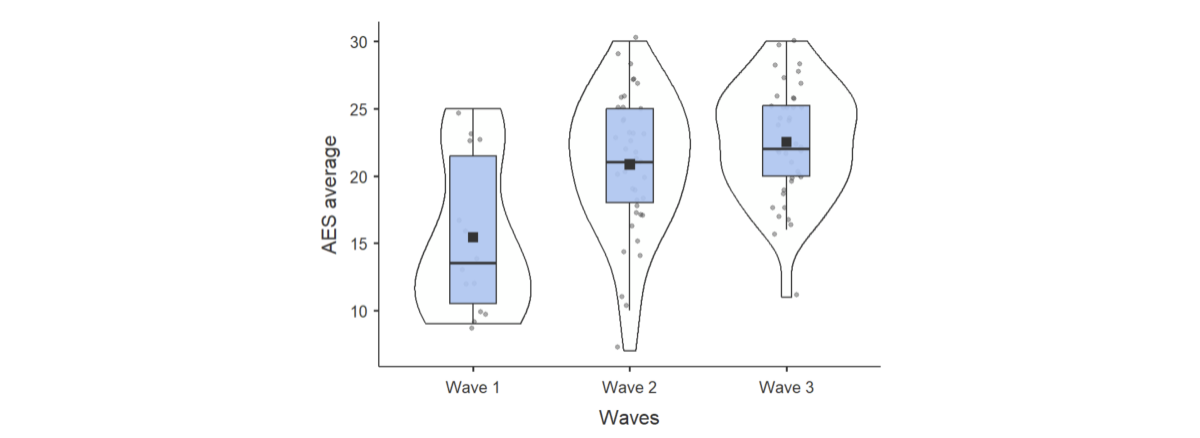
AES scores across the 3 experimental waves. AES: Acceptability E-scale.

The mean AES scores over the 3 waves of observation showed a positive progression between waves (Figure 3): 15.4/30 (SD 5.81) for wave 1, 20.9/30 (SD 5.25) for wave 2, and 22.5/30 (SD 4.23) for wave 3; this indicated a steady increase over time. Using the Kruskal-Wallis test, the mean AES scores showed a significant increase between waves (*χ*²_2_=13.4, *P*<.001). The Mann-Whitney test revealed no significant differences in AES scores between male and female participants (U=960, *P*=.71) and between patients and informal caregivers (U=905, *P*=.57).

**Figure 4:**
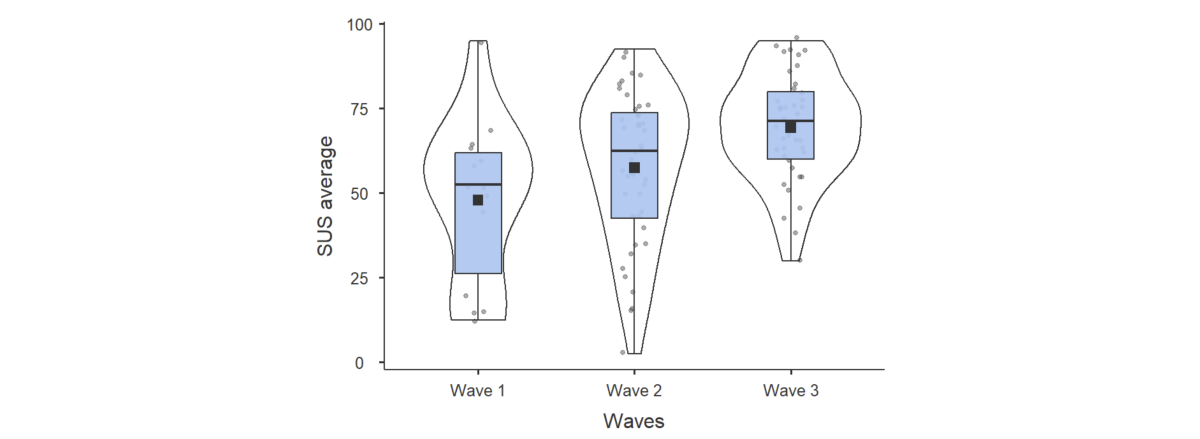
SUS scores across the 3 experimental waves. SUS: System Usability Scale.

Analysis of AES scores across the 3 experimental waves showed a significant difference between waves 1 and 2 (*P*<.01), as well as between waves 1 and 3 (*P*<.001). Additionally, no significant difference was observed between waves 2 and 3.

Based on these findings, an item-by-item analysis was conducted to identify specific differences across themes related to system acceptability. Tukey post hoc tests revealed significant differences in 4 of the 6 items: robot’s usability, robot’s perceived usefulness in the hospital setting, robot’s response time, and overall satisfaction with the robot.

Regarding satisfaction with the robot, a significant mean difference of –0.956 was observed between waves 1 and 2 (*P*=.02) and a difference of –1.336 between waves 1 and 3 (*P*<.001). With respect to the robot’s perceived usefulness in the hospital, a significant mean difference of –1.289 was found between waves 1 and 3 (*P*=.004). The robot’s response time showed a significant difference of –1.86 between waves 1 and 2 and a significant difference of –2.079 between waves 1 and 3 (*P*<.001). Finally, on overall robot satisfaction, a mean difference of –0.815 was observed between waves 1 and 2 (*P*=.042), as well as a mean difference of –1.246 between waves 1 and 3 (*P*<.001).

### System Usability Scale

The mean SUS scores over the 3 waves of observation showed a positive progression between waves: 47.9/100 (SD 24.18) for wave 1, 57.4/100 (SD 22.46) for wave 2, and 69.3/100 (SD 16.03) for wave 3; this indicated a steady increase over time. Using the Kruskal-Wallis test, the mean SUS scores showed a significant increase between waves (*χ*²_2_=11.4, *P*<.001). The Mann-Whitney test revealed no significant differences in SUS scores between men and women (U=848, *P*=.14) and between patients and informal caregivers (U=855, *P*=.19).

Analysis of SUS scores across the 3 experimental waves revealed significant differences between waves 1 and 3 (*P*<.01) and between waves 2 and 3 (*P*<.05). No significant difference was found between waves 1 and 2 (*P*>.05).

Based on these findings, an item-by-item analysis was conducted to examine specific differences in responses related to system usability. Tukey post hoc tests revealed significant differences in 5 of the 10 items: conversation complexity, assistance, inconsistency, ease of use, and user confidence.

Regarding the complexity of conversations, a significant difference of –1.089 was observed between waves 1 and 3 (*P*=.01). Regarding the assistance required to interact with the robot, a significant difference of –1.182 was observed between waves 1 and 3 (*P*=.02). Regarding the design of the robot’s functionality, a significant difference of –1.039 was observed between waves 1 and 3 (*P*=.005), as well as a mean difference of –0.569 between waves 2 and 3 (*P*=.04). Robot inconsistencies showed a significant difference of –1.28 between waves 1 and 2 (*P*=.003) and –1.632 between waves 1 and 3 (*P*<.001). Finally, regarding confidence using the robot, a significant difference –1.050 was observed between waves 1 and 3 (*P*=.01).

### Qualitative Data

#### Thematic Qualitative Analysis

A thematic qualitative analysis of participants’ feedback was conducted to explore perceptions of the ARI robot’s acceptability and usability. Verbatim responses from patients and informal caregivers were reviewed and categorized into 5 themes representing key aspects of user experience: (1) ease of use, (2) comprehension of the robot’s responses, (3) perceived usefulness of the robot in a hospital setting, (4) quality of interaction, and (5) overall system satisfaction. These themes correspond to established dimensions of acceptability (eg, perceived usefulness, satisfaction) and usability (eg, ease of use, comprehension, efficiency). Each theme is illustrated next with representative quotes translated from French to English.

#### Ease of Use

The robot’s ease of use, an essential component of acceptability, was progressively enhanced throughout the study. Although its physical ergonomics remained constant, updates to the dialogue system facilitated more fluid and coherent interactions. These improvements contributed to participants’ perceptions of the robot as simpler and more intuitive to operate. Several participants pointed out that the novelty of the robot could, at first, generate a certain amount of reluctance, suggesting that perceived ease of use also depended on experience acquired through interaction. For example, in wave 1, some found the voice interface practical:

The voice is helpful because I can’t see well [referring to the transcript on the robot’s screen]. You just have to ask it a question.

Informal caregiver, wave 1

Others found it unintuitive:

Not at all [easy to use]!

Patient, wave 1

However, some participants pointed out the limit of novelty when faced with a robot:

I mean, we're not used to it. When you're used to it, it's easy, but not the first time. It's a bit confusing. You have to get used to it.

Patient, wave 1

This need for an adaptation period was most prominent in wave 1, when the system was less refined, but it also emerged in a few cases in waves 2 and 3. This suggests that although improved usability facilitated smoother interactions, user familiarity remained a factor influencing initial comfort in engaging with the robot.

As waves progressed, more participants acknowledged the simplicity of interacting with the robot:

It's easy...you just need to ask questions, and if the question is relevant, it answers.

Patient, wave 2

However, some participants reported challenges in formulating their questions appropriately:

I had to force myself to phrase my questions differently from how I would naturally say them.

Informal caregiver, wave 3

#### Understanding the Robot’s Responses

Although voice synthesis remained consistent, updates to the dialogue system, particularly the integration of an LLM facilitated clearer and more coherent exchanges. Participants generally found the language comprehensible; however, some reported mismatches between their questions and the robot’s responses, suggesting that perceived comprehension remained sensitive to the contextual specificity of the interactions. In wave 1, participants’ opinions ranged from finding the robot’s speech fully understandable (“They were completely understandable and easy” [patient, wave 1]) to reporting inconsistencies in comprehension. In wave 2, many agreed that the robot spoke clearly:

It was perfectly understandable.

Patient, wave 2

However, some responses were found to be off-topic:

Its explanations are clear, but some responses were off the mark.

Informal caregiver, wave 2

In wave 3, participants generally found the language clear:

I found it very clear and precise.

Patient, wave 3

However, some noted occasional mismatches between their questions and the robot’s responses:

Not entirely understandable, because it made me ask the same question multiple times.

Patient, wave 3

#### Perceived Usefulness of the Robot in a Hospital Setting

Perceptions of the robot’s usefulness evolved over the 3 waves. Although some participants found it useful for providing basic information, others expected a more interactive experience. Over time, the focus shifted to the accuracy of the robot’s responses, with some seeing it as a “glimpse into the future,” while others emphasized its current limitations. In wave 1, some found it practical for basic information:

Just telling me [the location needed] it's next door was enough for me.

Informal caregiver, wave 1

However, others expected a more interactive experience:

If it [the robot] had come with me [to show me the path], it would have been better.

Patient, wave 1

In wave 2, usefulness was conditional on the accuracy of responses:

Plenty useful, but only if it understood the question and answered it correctly.

Informal caregiver, wave 2

It did the job, but a good signpost could do it too.

Informal caregiver, wave 2

By wave 3, opinions remained divided, with some participants perceiving the robot as indicative of future technological developments:

It’s an opening into something futuristic. So, it’s useful to me.

Informal caregiver, wave 3

However, for others, opinions remained mixed with respect to the robot:

Well, it annoyed me quite a bit [...] Every time I ask it a question, it tells me to go to the reception.

Informal caregiver, wave 3

Some participants highlighted its efficiency and consistency compared to human interactions:

It answered all those questions 100%. [Informal caregiver, wave 3)

Even if some people might find a human more pleasant, humans can get tired or irritated—whereas ARI [the robot] never does.

Informal caregiver, wave 3

#### Quality of Interaction

Throughout the study, updates to the robot enhanced the fluidity of interactions and the quality of user feedback—2 factors critical to its acceptability. These improvements, particularly in the response speed, interruption management, and response richness, were reflected in participants’ feedback. During wave 1, the robot demonstrated inconsistent response patterns, as reported by participants:

I asked a question, but it [the robot] responded with something completely unrelated—it was totally incoherent.

Patient, wave 1

Some participants also perceived that the robot had a limited range of programmed responses:

I have the impression that it is programmed only to answer certain questions.

Patient, wave 1

Additionally, participants noted that the robot often repeated suggestions unrelated to their queries:

But it does offer to go to lunch a lot.

Patient, wave 1

In wave 3, some participants noted that the robot responded promptly:

You ask the question, and it answers almost immediately.

Informal caregiver, wave 3

However, others reported challenges in interrupting the robot while it was delivering a response:

You can't interrupt it, and that bothered me a bit.

Informal caregiver, wave 3

Furthermore, some participants expressed a preference for the robot’s responses over those provided by human receptionists:

I prefer your robot’s answers to those of the ladies at the reception.

Patient, wave 3

There are humans who aren't as direct and precise [...] I'm surprised.

Patient, wave 3

#### General Satisfaction

Overall satisfaction with the experimentation of the robot reflected growing acceptance in terms of both functional effectiveness and ease of use, with clear progression observed from the first to the final wave. In wave 1, some participants appreciated the robot’s novelty:

I think it is really nice. I think it's fun and funny.

Informal caregiver, wave 1

I like it—it’s nice. Really nice. It’s great to see a robot. But a human is still nicer.

Informal caregiver, wave 1

Conversely, some participants expressed dissatisfaction:

I’m sorry if I sound upset, but I didn’t feel any satisfaction.

Patient, wave 1

I didn’t get any satisfaction—the robot didn’t help me at all. It’s actually a bit scary.

Patient, wave 1

In wave 2, reactions remained mixed; some participants found the robot engaging and entertaining:

I find it amazing, it’s super.

Patient, wave 2

It's impressive, it's the first time it's happened to me, I didn't know robots at all before today, and it's impressive to see a machine that you ask questions to, that answers you.

Patient, wave 2

However, others highlighted its limitations:

I’m sure the robot has potential, but at the moment, it’s not performing well.

Informal caregiver, wave 2

In wave 3, participant feedback was generally more favorable. Participants described the robot as “very interesting, very instructive because it’s very knowledgeable. It answered me perfectly well” (patient, wave 3) and declared that “it’s funny because you don’t expect to find something like this, and it does have quite a lot of answers” (informal caregiver, wave 3). Others expressed an overall appreciation:

Oh, I like it a lot. I think it's nice, it has beautiful eyes.

Patient, wave 3

I like this thing.

Patient, wave 3

## Discussion

### Principal Findings

This study provides one of the first in situ evaluations of a SAR equipped with an LLM in a geriatric hospital setting. Quantitative data showed a significant increase in both acceptability and usability of the ARI robot, corroborating the qualitative findings. Participants reported improved ease of use, comprehension, and perceived usefulness across experimental waves, as reflected in the significant increases in AES and SUS scores. Acceptability scores increased from 15.4/30 in wave 1 to 22.5/30 in wave 3 (*P*<.001), while usability scores rose from 47.9/100 to 69.3/100 (*P*=.003). Verbatim feedback reflected a growing appreciation for the robot’s functionality and ease of interaction, aligning with observed improvements in satisfaction and perceived usefulness. These improvements are largely attributable to technical upgrades, most notably the integration of an LLM, which enhanced the naturalness, contextual relevance, and consistency of interactions. The experimental conversational system, developed collaboratively within the European H2020 SPRING project [58], consists of an audiovisual perception pipeline that captures speech and behavioral cues, processed by a dialogue system powered by an LLM to synthesize responses into sound. Collectively, these developments contributed to a more fluid, engaging, and user-centered experience with the robot.

Although the ARI robot is commercially available, the conversational system developed in this study remains experimental. The final scores did not meet established thresholds for acceptability (AES score>25.81) and usability (SUS score>72), underscoring the need for further refinement prior to large-scale hospital implementation, such as transitioning to full-duplex communication, adding multilingual support, and incorporating nonverbal feedback. Nonetheless, the upward trajectory of these scores is encouraging, suggesting strong potential for future clinical deployment as performance approaches benchmark criteria.

### Limitations

This study presents several limitations related to both the experimental design and the technical capabilities of the robotic system.

From a methodological perspective, the study was conducted at a single site within an innovation-friendly clinical environment. Although this facilitated implementation, it may limit the generalizability of the findings to institutions with different organizational structures or cultural contexts. Moreover, the recruitment strategy relied on voluntary participation, introducing a potential self-selection bias.

Another limitation concerns the ecological validity of the experimental conditions. Although the ARI robot was deployed in a hospital setting, the interactions took place in a quiet, controlled room rather than in a dynamic waiting area. This may have minimized external distractions and reduced communication challenges typically present in real-world hospital contexts. In addition, the interaction occurred only once per participant. As a result, the findings largely reflect first impressions and do not capture how user perceptions evolve with repeated exposures or long-term use. Longitudinal studies are needed to assess the durability of engagement and trust over time.

On a technical dimension, although the integration of an LLM improved conversational coherence, the system remained prone to occasional misinterpretations or generic responses, especially when confronted with vague or ambiguous input. This limitation is consistent with known issues in LLM-based dialogue systems, which often struggle with generating contextually appropriate and nongeneric responses when input is vague or underspecified [59]. Furthermore, the robot’s speech recognition system showed sensitivity to user-specific features, such as accent, the speech rate, and background noise. Finally, the absence of multilingual support reduced inclusiveness for non–French-speaking users, an important consideration in multicultural health care environments.

### Comparison With Prior Work

Our findings align with the existing literature showing that AI-powered SARs can significantly enhance user engagement when interactions are intuitive and socially appropriate [5,19,26]. The integration of an LLM between waves 1 and 2 notably improved the ARI robot’s conversational fluency and contextual relevance, features widely recognized as essential for effective HRI [1,2]. Although natural language processing has long been identified as a key component of user engagement, few studies have directly captured the impact of integrating an LLM into SARs in a health care context. Most prior evaluations were either conducted before the emergence of transformer-based language models (pre-2019) or focused on single-instance deployments with fixed systems. Moreover, although most existing studies on SARs have been conducted in controlled environments, such as laboratories or nursing homes [3,4], few have examined how real-time system refinements affect user perceptions over successive implementation phases. This study addressed this gap by documenting how progressive improvements to the LLM-based dialogue system influence usability and acceptability in situ over 3 successive waves.

Future systems should integrate multimodal strategies (eg, gesture recognition, facial expression analysis, affective cues) to support more natural and empathetic communication in health care environments [16,49].

Our results also align with previous research advocating for voice-based interaction as particularly suitable for OAs, especially in contexts where digital literacy may vary [22,23]. Participants consistently favored spoken communication over touchscreen input. Furthermore, the inclusion of real-time transcription improved accessibility, confirming prior findings on the value of combining auditory and visual cues for inclusive design [60].

Confidence in using the system was closely linked to interaction quality as although wave 3 participants, who received consistent and context-aware responses, gained confidence, those in earlier waves, confronted with erratic outputs, voiced concerns about the robot’s reliability. Interestingly, some participants also blamed themselves, reflecting a well-documented phenomenon in HRI literature, automation bias, where users tend to overestimate the capabilities of intelligent systems and assume personal responsibility for communication failures [39,61]. These findings underscore the critical role of transparency and predictability in AI-driven behaviors as key determinants of user confidence, reinforcing prior research in HRI [16,62,63].

Finally, our results reaffirm the need for user familiarization with voice-based robotic interaction in health care contexts. For the majority of participants, this study marked their first direct interaction with a SAR, and many indicated the need for a period of adjustment. Initial hesitation often gave way to positive engagement, echoing previous research showing that structured onboarding and repeated exposure are key to long-term acceptability [11].

### Recommendations

Drawing on the empirical findings of this study, the following recommendations are intended to inform the design and implementation of SARs tailored to the specific interactional needs of OAs in geriatric care settings. These evidence-based insights aim to guide researchers and industry stakeholders in developing socially assistive technologies that are both user centered and contextually appropriate within health care environments.

#### Improve Natural Language Processing, Speech Recognition, and Multilingual Support

To enhance the quality of HRI in geriatric care, it is recommended that future SARs integrate more advanced natural language processing capabilities than those available in current open models to ensure greater coherence and contextual relevance in responses. Speech recognition systems should be optimized to account for prosodic variation, regional accents, and articulation differences (eg, slower speech rate, reduced vocal intensity, hesitations, or imprecise consonant production) commonly observed among OAs due to age-related changes in respiratory and orofacial motor control [64]. In addition, the incorporation of multilingual functionality is essential to accommodate the linguistic diversity of users in health care settings and to promote equitable access to robotic services.

#### Develop Clear Onboarding Protocols and Autonomous Robot Self-Presentation

To facilitate user engagement and support initial interaction, it is recommended that future deployments of SARs include a structured onboarding protocol. This may involve brief demonstrations or the distribution of printed materials (eg, leaflets with example questions) to familiarize users with the robot’s capabilities. However, recognizing that real-world hospital settings may not consistently offer human assistance at the point of interaction, SARs should also be equipped with a robust self-introduction feature. This feature should autonomously guide users through the robot’s purpose, functionalities, and appropriate use cases, clarifying when and how to engage and what types of questions it can address. Such improvements are essential for promoting user autonomy, reducing uncertainty, and improving the perceived usefulness of SARs in health care environments.

Such improvements are essential for OAs, who often face reduced digital literacy, cognitive decline or impairments, or heightened anxiety when interacting with unfamiliar technologies. Research has shown that guided introductions, whether verbal or visual, enhance initial confidence, reduce hesitation, and support sustained engagement [65,66].

#### Ensure Compliance With Privacy, Ethical, and Regulatory Standards

It is essential that the development and deployment of SARs in health care settings align with existing ethical guidelines and data protection regulations. Particular attention must be given to the collection, processing, and storage of sensitive health-related information. Developers and implementers should establish clear protocols for obtaining informed consent, ensuring transparency regarding data usage, and enabling users to understand the scope and limitations of data handling. Additionally, SARs should be designed to support confidential interactions, especially when used with vulnerable populations, such as OAs, by incorporating features that safeguard user privacy and uphold professional standards of care. Early integration of ethical and legal considerations will be critical to building trust and ensuring responsible implementation.

#### Enhance Interruption Management and Conversational Fluidity

Participants highlighted the difficulty of interrupting the ARI robot while it was speaking, a limitation that disrupted the natural flow of dialogue. To address this issue, future iterations of SARs should improve turn-taking mechanisms and enable responsive interruption handling, especially in multiparty or fast-paced care environments. These refinements are essential for supporting user-led interaction, minimizing frustration, and improving conversational naturalness.

#### Enable Autonomous Navigation and Spatial Guidance

Several participants expressed the desire for the ARI robot to not only provide verbal instructions but also physically accompany them to their destination. Incorporating autonomous navigation capabilities would expand the robot’s role from an informational assistant to an active guide, offering greater support to users with mobility or orientation difficulties in complex care environments, such as hospitals. To ensure safe and effective guidance, particular attention should be paid to obstacle detection and navigation in dynamic environments, as well as to adapting the robot’s speed to the walking pace of OAs.

### Conclusion

This study showed that SARs, when iteratively enhanced and evaluated in real-world conditions, can reach growing levels of acceptability and usability among OAs and their informal caregivers. The integration of an LLM was a turning point in improving the ARI robot’s ability to engage in meaningful, coherent interactions. These findings suggest that SARs, when designed with user needs in mind, hold strong potential for supporting care delivery in geriatric hospital settings.

Future work should focus on further leveraging the capabilities of LLMs to enable adaptive, personalized interactions that respond to individual users’ communication styles, memory, and emotional cues. Additionally, the integration of multimodal communication features, such as gaze tracking, gesture and facial expression recognition, and targeted dialogue strategies, will be essential to enhancing the ARI robot’s relevance, trustworthiness, and effectiveness in complex health care environments.

## Appendix

Multimedia Appendix 1 Evaluation scales: AES and SUS (translated from French to English). AES: Acceptability E-scale; SUS: System Usability Scale.

## Abbreviations

AES: Acceptability E-scale
AI: artificial intelligence
DCH: day care hospital
HRI: human-robot interaction
LLM: large language model
MMSE: Mini-Mental State Examination
OA: older adult
SAR: socially assistive robot
SUS: System Usability Scale

## Data Availability

Requests to access the datasets should be sent to the corresponding author.
